# Simple method for calculation of the frequency of standardisation of analytical measurements

**DOI:** 10.1155/S1463924680000557

**Published:** 1980

**Authors:** J. Inczédy

**Affiliations:** Institute of Analytical Chemistry University of Chemical Engineering Veszprérn Hungary


### Conclusions

A need has been indicated for more attention to be given to achieving compatible measurements from automated instruments. It is argued that repeatability is not a sufficient criterion for assessing the reliability of analytical methods and instruments. Co-operation is required between manufacturers, their customers and suppliers of certified reference materials, to ensure the necessary work to achieve compatibility is undertaken in good time to meet the needs at a realistic cost.

ACNOWLEDGEMENTS

I would like to thank my colleagues at the National Physical Laboratory for useful comments on the content of this paper.

REFERENCES

[1] Uriano, G. A. and Gravatt, C. C., CRC Critical Reviews in Analytical Chemistry, October 1977, 361.

[2] Cox, J. D. and Ridsdale, P. D., NPL Report Chem 93, 1978.

### Simple method for calculation of the frequency of standardisation of analytical measurements

**J Inczédy**

*Institute of Analytical Chemistry, University of Chemical Engineering, Veszprém, Hungary.*

### Introduction

In analytical chemical measurements, when measurements are periodically repeated or the measurement itself takes a long time, for example in process control, the most significant source of error is the long term instability of the instrument. In instrumental analytical measurements, the precision of the measurement is often acceptable although the bias can be as much as 20–50% due to the shift of the zero point of the instrument. In many cases, the latter problem is not considered by the analyst because it is one which is not easily grasped.

In this paper a simple method is presented by which the approximate frequency of standardisation of the instrument can be calculated.

Recently, several papers have appeared in the literature dealing with the acceptable total error (that is, both systematic and random errors) of analytical measurements [1 and 2]. Also, a method of eliminating the error caused by the shift of the zero point using a linear interpolation has been described [3].

### Calculation of the time interval between standardisations

For the calculation of the optimum time interval between two standardisations, the following assumptions and considerations are made:-

(i) That there is a steady state zero point migration which can be approximated by a periodic sine wave function.

(ii) That the shift between and during the two standardisation procedures can be approximated with a linear function.

(iii) That the distribution of the results of measurements is of a normal Gaussian form.

(iv) That the standardisation must be repeated when the error arising from the migration becomes significant in relation to the standard deviation of the measurements.

The first assumption was made because the slow migration of the zero point of the instrument in most cases is periodic and the time of the period depends on the periodic changes of the environment or electrical mains load.

The second assumption is also valid since the time interval between the standardisation points is usually much smaller than the time of the period.

In analytical measurements, the third assumption is accepted.

The fourth assumption is made because the difference between a measured value obtained as the mean of close successive measurements and another one will be significant when the limit of 2s is passed. ('s' is the standard deviation of the 'parallel measurements', carried out during a short time interval $\tau$).

Between the two successive standardisations the error caused by the slow migration of the zero point can be taken into consideration when assessing the true measurements by linear interpolation if the migration rate is known. This is possible only in those cases where very rigorous measurements are necessary.

Figure 1 shows a diagram of the zero point migration along with two idealised curves. The original curve can be approximated by a sine wave function or by an equivalent regular triangle function. The sine wave function and the triangle function are equivalent when their period time T and variance $\sigma^2$ are the same.

According to the calculations, the sine and triangle functions exhibit the same variance when their amplitudes are in the following relation:

$$a = 0.8246\ A \qquad (1)$$

a is the amplitude of the sine wave function, and A is the peak amplitude of the triangle function. Their variance may be expressed as follows :

$$\sigma^2 = \frac{a^2}{2} \qquad (2)$$

A small idealised part of Figure 1 is shown in greater detail in Figure 2, where the curve is approximated with a straight line. During and after the setting of the zero point the shift is increasing linearly. By parallel measurements the setting can be made with confidence, but at the expense of a definite time consumption $\tau$. When the shift value reaches the 2s (twice the standard deviation of the closely successive measurements) the standardisation or zero point setting procedure is recommenced.

To calculate the time interval between two standardisations $t_{st}$, the proportionality rule of the similar rectangular triangles can be applied (Figure 2).

$$\frac{2s}{A} = \frac{t_{st} - \frac{\tau}{2}}{T/4} \qquad (3)$$

using equation (1) and (2), we obtain:

$$t_{st} = 0.29\frac{s}{\sigma}T - \frac{\tau}{2} \qquad (4)$$

If $\tau \ll t_{st}$, the last term can be omitted.

$$t_{st} = 0.29\frac{s}{\sigma}T \qquad (5)$$

or in frequency form:

$$f_{st} = 3.43\frac{\sigma}{s}f_d \qquad (6)$$

where $f_{st} = \frac{1}{t_{st}}$ is the frequency of the zero point setting or standardisation, while $f_d = \frac{1}{T}$ is the frequency of the fluctuation of the zero point.

Equation (6) shows that the standardisation frequency depends on the ratio of the standard deviation of the drift and that of the instrumental measurements. It also depends on the low frequency of the drift motion.

However the complete standardisation of an analytical method cannot be restricted to the setting of the zero point, or setting of the relative position of the analytical curve. Control and adjustment of the slope of the analytical curve is also necessary. Analytical experience shows, however, that the errors originating from the drift are usually much larger than those originating from the changes of the slopes of the analytical curves (or distortion) emerging in time.

The formulae deduced above were varified using experimental data.

### Experimental validation

The following experiment was carried out at a typical example. A liquid chromatograph consisting of a M 6000 pump (Waters Association Inc, USA), a thermostatted (30°C) column packed with Nucleosil C-18 and a Varian UV detector (254 nm) type LC-4020 were run continuously overnight. An aqueous eluent containing 25% MeOH was pumped at a flow rate of 2 ml/min. The detector trace was recorded at a sensitivity of 0.04 absorbance units full scale (25 cm); chart speed was 0.2 cm/min.

Parts of the curve which was obtained are shown schematically in Figure 3. This approximates to a sine wave. The total period, including first a concave and later a convex part, was

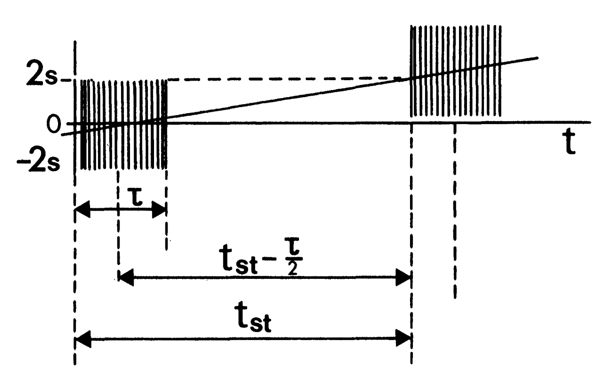

*Figure 2. Determination of the time interval of the standardisation: s standard deviation of the measurements, $\tau$ time of the standardisation, $t_{st}$ time interval between two standardisations. (The slope of the line 4 A/T).*

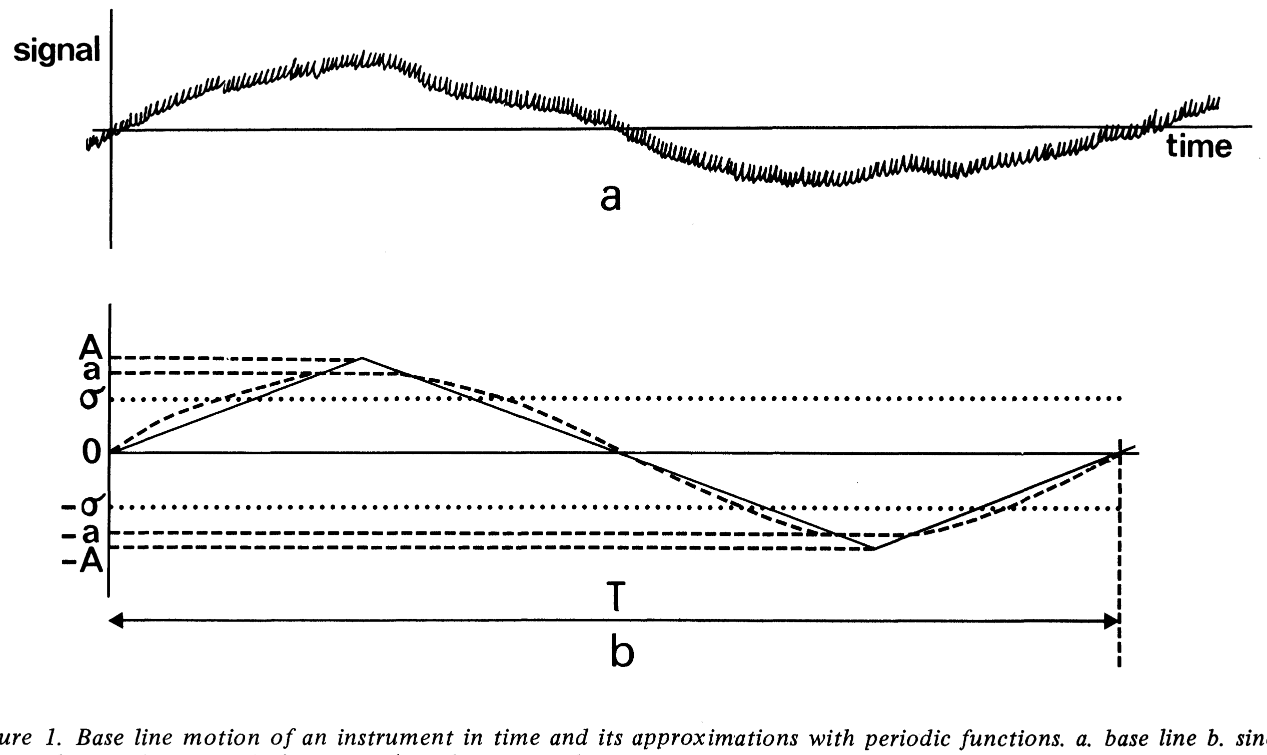

*Figure 1. Base line motion of an instrument in time and its approximations with periodic functions. a. base line b. sine wave and equivalent triangle functions. (a and A are amplitudes, σ standard deviation, T time of the period).*

estimated as 7.5 hours. The amplitude a (defined as half of the difference between the deepest and highest parts), was 5 mm. The distance of the two parallel contour lines of the noisy trace was 3 mm. The distance between the contour lines corresponds approximately to 5s, which gives a value for s of 0.6 mm.

The variance of the migration is given by:

$$\sigma^2 = \frac{a^2}{2} = \frac{5^2}{2} = 12.5$$

and

$$\sigma = 3.54 \text{ (mm)}$$

The shortest time interval between the two successive settings (if the time of the setting can be neglected) is therefore given by:

$$t_{st} = 0.29\frac{0.6}{3.54}\ 450 = 22 \text{ (min)}$$

Practically, this means that after each chromatographic separation the zero point must be reset. This observation fully agrees with the author's experience.

Thus to calculate the frequency of standardisation, the amplitude (a) and the periodic time (T) of the zero point migration from data of long term experiments must be determined. From the amplitude the standard deviation ($\sigma$) is then calculated using equation (2). If the precision (s) of the single measurements, and $\sigma$, T, and $\tau$ are known, the necessary time interval $t_{st}$ can be calculated using equation (4) or (5).

### Conclusion

The principle of the method of the calculation can be used also in those cases where the 'drift' or the change of the systematic error does not originate from the instability of an instrument, but from other sources. The change, however, must be periodic.

REFERENCES

[1] McFarren, E. F., Lishka, R. I. and Parker, J. H., Analytical Chemistry, 1970, **42**, 358.

[2] Midgley, D., Analytical Chemistry, 1977, **49**, 510.

[3] Gegus, E., Paper at the 10. Spektrometertagung, den Hagg 1974.

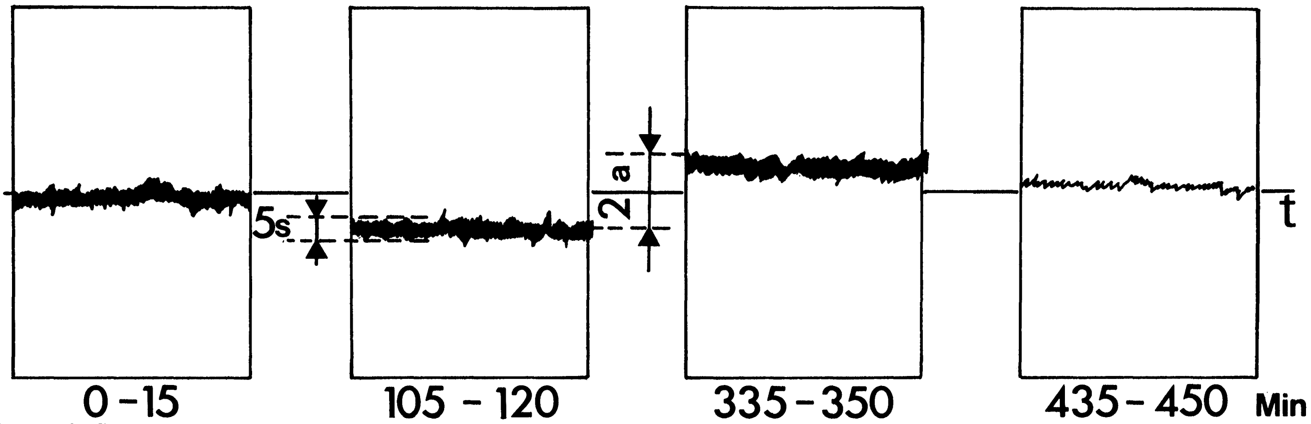

*Figure 3. Segments of an original record obtained by a liquid chromatograph. Evaluation of the parameters used for calculation.*

### Notes for Contributors

### Presentation of manuscripts

Manuscripts should be typed (double-spaced) on one side of the paper only and with generous margins. The title should be brief and informative avoiding the word "new" and its synonyms. The full list of authors with their affiliations and full address(es) should appear on the title page. On a separate sheet an abstract of no more than 150 words is required. This should succinctly describe the scope of the contribution and highlight significant findings or innovations. It should be written in a style which can easily be translated into French and German.

*The Concise Oxford Dictionary* and Fowler's *Modern English Usage* (both published by Oxford University Press) should be used as the standard for spelling and grammar. Abbreviations should be limited to those generally recognised, or where a frequently occuring term is abbreviated it should, in the first instance, be explained thus "flow injection analysis (FIA) ..." and the abbreviation used thereafter. Abbreviations, for standard measures and units should follow SI recommendations. There are various publications giving guidance on the use of SI units.

References should be indicated in the text by numerals following the author's name, i.e. Skeggs [6]. On a separate sheet of paper, list all references in numerical order thus:

[6] Skeggs, L.T., *American Journal of Clinical Pathology*, 1959, **28**, 311.

Note that journal titles are given in full. Where there is more than one author, the form Foreman et al. should be used in the text but all authors should be named in the list of references. When reference is made to a chapter in a book the reference should take the following form:

[7] Malmstadt, H.V. in "Topics in Automatic Chemistry" Ed. Stockwell P.B. and Foreman J.K. 1978 Horwood, Chichester, pp. 68-70.

Only work which has been published or has been accepted for publication should be cited. Avoid the citation of documents which are subject to restricted circulation, patent literature, unpublished work and personal communications. The latter can be mentioned in the text in parenthesis.

To illustrate a paper line diagrams are preferred to photographs. Photographs should only be used when they significantly add to the discussion. Diagrams, charts and graphs should be carefully drawn in black ink on stout card or heavy quality tracing paper. Most illustrations are reduced for publication; to allow for this originals should be between 16 and 36 cm wide (the depth must not exceed 50 cm). The lettering of diagrams should be sufficiently clear to withstand reduction. Except in the case of proper names, all lettering should be in lower case print. If photographs are used they must be supplied in the form of clear, unmounted, glossy, black and white prints. "Instant" photographs are not normally acceptable. All illustrations must be identified on the reverse showing the figure number and the author's name.

Each illustration should have a fully explanitory caption. Captions should be typed together on a separate sheet of paper; they must *not* be an inseparable part of the illustration.

